# Psychology, replication & beyond

**DOI:** 10.1186/s40359-016-0135-2

**Published:** 2016-06-01

**Authors:** Keith R. Laws

**Affiliations:** School of Life and Medical Sciences, University of Hertfordshire, Hatfield, UK

## Abstract

Modern psychology is apparently in crisis and the prevailing view is that this partly reflects an inability to replicate past findings. If a crisis does exists, then it is some kind of ‘chronic’ crisis, as psychologists have been censuring themselves over replicability for decades. While the debate in psychology is not new, the lack of progress across the decades is disappointing. Recently though, we have seen a veritable surfeit of debate alongside multiple orchestrated and well-publicised replication initiatives. The spotlight is being shone on certain areas and although not everyone agrees on how we should interpret the outcomes, the debate is happening and impassioned. The issue of reproducibility occupies a central place in our whig history of psychology.

In the parlance of Karl Popper, the notion of *falsification* is seductive – some seem to imagine that it identifies an *act* as opposed to a *process*. It often carries the misleading implication that hypotheses can be readily discarded in the face of something called a ‘failed’ replication. Popper [46] was quite transparent when he declared *“…*a few stray basic statements contradicting a theory will hardly induce us to reject it as falsified. We shall take it as falsified *only if we discover a reproducible effect which refutes the theory*. In other words, we only accept the falsification if a low level empirical hypothesis which describes such an effect is proposed and corroborated.” (p.203: my italics). Popper’s view might reassure those whose psychological models have recently come under scrutiny through replication initiatives. We cannot, nor should we, close the door on a hypothesis because a study fails to be replicated. The hypothesis is not *nullified* and ‘nay-saying’ alone is an insufficient response from scientists. Like Popper, we might expect a testable alternative hypothesis that attempts to account for the discrepancy across studies; and one that itself may be subject to testing rather than merely being *ad hoc*. In other words, a ‘failed’ replication is not, in itself, the answer to a question, but a further question.

## Replication, replication, replication

At least two key types of replication exist: *direct* and *conceptual.* Conceptual replication generally refers to cases where researchers ‘tweak’ the methods of previous studies [43] and when successful, may be informative with regard to the *boundaries* and possible moderators of an effect. When a conceptual replication fails, however, fewer clear implications exist for the original study because of likely differences in procedure or stimuli and so on. For this reason, we have seen an increased weight given to *direct* replications.

How often do direct and conceptual replications occur in psychology? Screening 100 of the most-cited psychology journals since 1900, Makel, Plucker & Hegarty [40] found that approximately 1.6 % of all psychology articles used the term *replication* in the text. A further more detailed analysis of 500 randomly selected articles revealed that only 68 % using the term *replication* were actual replications. They calculated an overall replication rate of 1.07 % and Makel et al. [40] found that only 18 % of those were direct rather than conceptual replications.

The lack of replication in psychology is systemic and widespread, and particularly the bias against publishing direct replications. In their survey of social science journal *editors*, Neuliep & Crandall [42] found almost three quarters preferred to publish novel findings rather than replications. In a parallel survey of *reviewers* for social science journals, Neuliep & Crandall [43] found over half (54 %) stated a preference for new findings over replications. Indeed, reviewers stated that replications were “Not newsworthy” or even a “Waste of space”. By contrast, comments from natural science journal editors present a more varied picture, with comments ranging from “Replication without some novelty is not accepted” to “Replication is rarely an issue for us…since we publish them.” [39].

Despite an enduring historical abandonment of replication, the tide appears to be turning. Makel et al. [40] found that the replication rate after the year 2000 was 1.84 times higher than for the period between 1950 and 1999. In a more recent evolution, several large-scale direct replication projects have emerged during the past 2 years including: the *Many Labs project* [33]; a set of preregistered replications published in a special issue of *Social Psychology* (Edited by [44]); the *Reproducibility Project of the Open Science Collaboration* [45]; and the *Pipeline Project* by Schweinsberg et al. [50]. In two of these projects (Many Labs by [33]; Pipeline Project by [50]), a group of researchers replicated samples of studies, with each group replicating all studies. In the two remaining projects, a number of research groups each replicated one study, selected from a sample of studies (Registered Reports by [44]; Open Science Collaboration, [45]). Each project ensured that replications were sufficiently powered (typically in excess of 90 % -thus offering a very good probability of detecting true effects) and where possible, used the original materials and stimuli as provided by the original authors. It is worth considering each in more detail.

*Many Labs* involved 36 research groups across 12 countries who replicated 13 psychological studies in over 6,000 participants. Studies of classic and newer effects were selected partly because they had simple designs that could be adapted for online administration. Reassuringly perhaps, 10 of the 13 effects replicated consistently across 36 different samples with, of course, some variability in the effect size reported compared to the original studies – some smaller but also some larger. One effect received weak support. Only two studies consistently failed to replicate and both involved what are described as ‘*social priming’* phenomena. One study where ‘accidental’ exposure to a US flag resulted in increased conservatism amongst Americans [11]. Participants viewed four photos and were asked to just estimate the time-of-day in the photo – the US flag appeared in two photos. Following this, they completed an 8-item questionnaire assessing their views toward various political issues (e.g., abortion, gun control). In the second priming study, exposure to ‘money’ had resulted in endorsement of the current social system [12]. In this study, participants completed demographic questions against a background that showed a faint picture of US $100 bills or the same background but blurred. Each of these two priming experiments had a single significant *p*-value (out of 36 replications) and for flag priming, it was in the opposite direction to that expected.

Turning to the special issue of *Social Psychology* edited by Nosek & Lakens [44]. This contained a series of articles replicating *important* results in social psychology. *Important* was broadly defined as “…often cited, a topic of intense scholarly or public interest, a challenge to established theories), but should also have uncertain truth value (e.g., few confirmations, imprecise estimates of effect sizes).” One might euphemistically describe the studies as *curios*. The articles were first submitted as *Registered Reports* and reviewed prior to data collection, with authors being assured their findings would be published regardless of outcome, as long as they adhered to the registered protocol. Attempted replications included the “Romeo and Juliet effect” – does parental interference lead to increases in love and commitment (Original: [17]; Replication: Sinclair, Hood, & Wright, [53]), does experiencing physical warmth (warm therapeutic packs) increase judgments of interpersonal warmth (Original: [58]; Replication: Lynott, Corker, Wortman, Connell, Donnellan, Lucas, & O’Brien, [38]), does recalling unethical behavior lead participants to see the room as darker (Original: [3]; Replication: [10]); does physical cleanliness reduce the severity of moral judgments (original : [49]: [28]). In contrast to high replication rate of *Many Labs*, the *Registered Reports* replications failed to confirm the results in 10 of 13 studies.

In the largest crowdsourced effort to date, the *OSC Reproducibility project* involved 270 collaborators attempting to replicate 100 findings from 3 major psychology journals *Psychological Science (PSCI), Journal of Personality and Social Psychology (JPSP), and Journal of Experimental Psychology: Learning, Memory, and Cognition (JEP: LMC).* While 97 of 100 studies originally reported statistically significant results, only 36 % of the replications did so with a mean effect size of around half of that reported in the original studies.

All of the journals exhibited a large reduction of around 50 % in effect sizes, with replications from JPSP particularly affected - shrinking by 75 % from 0.29 to 0.07. The replicability in one domain of psychology (good or poor) in no way guarantees what will happen in another domain. One thing we know from this project, is that “…reproducibility was stronger in studies and journals representing cognitive psychology than social psychology topics. For example, combining across journals, 14 of 55 (25 %) of social psychology effects replicated by the *P* < 0.05 criterion, whereas 21 of 42 (50 %) of cognitive psychology effects did so.” The reasons for such a difference are debatable, but provide no licence to either congratulate cognitive psychologists or berate social psychologists. Indeed, the authors paint a considered and faithful picture of what their findings mean when they conclude “…how many of the effects have we established are true? Zero. And how many of the effects have we established are false? Zero. Is this a limitation of the project design? No. It is the reality of doing science”. (Open Science Collaboration p.4716-7) 

The studies that were not selected for replication are informative – they were described as “…deemed infeasible to replicate because of time, resources, instrumentation, dependence on historical events, or hard-to-access samples… [and some] required specialized samples (such as macaques or people with autism), resources (such as eye tracking machines or functional magnetic resonance imaging), or knowledge making them difficult to match with teams”. Thus, the main drivers of replication are often economic in terms of time, money and human investment. High cost studies are likely to remain castles in the air, leaving us with little insight about replicability rates in some areas such as functional imaging (e.g. [9]), clinical and health psychology (see Coyne, this issue), and neuropsychology.

The ‘*Pipeline project’* by Schweinsberg et al. [50] intentionally used a non-adversarial approach. They *crowdsourced* 25 research teams across various countries to replicate a series of 10 *unpublished* moral-judgment experiments from the lead author’s (Uhlmann) lab i.e., in the pipeline. This speaks directly to Lykken’s [37] proposal from nearly 50 years ago that “…ideally all experiments would be replicated before publication” although at that time, he deemed it ‘impractical’.

*Pipeline* replications included: the *Bigot–misanthrope effect* – whether participants judge a manager who selectively mistreats racial minorities as a more blameworthy person than a manager who mistreats all of his employees; *Bad tipper effect* - are people who leave a full tip, but entirely in pennies judged more negatively than someone who leaves less money, but in notes; the *Burn-in-hell effect* – do people perceive corporate executives as more likely to burn in hell than members of social categories defined by antisocial behaviour, such as vandals. Six of ten findings replicated across all of their replication criteria, one further finding replicated but with a significantly smaller effect size than the original, one finding replicated consistently in the original culture but not outside of it (*bad tipper* replicated in US and not outside), and two findings effects were unsupported.

The headline replication rates differed considerably across projects – occurring more frequently for Many Labs (77 %) and the Pipeline Project (60 %) than Registered Reports (30 %) and the Open Science Collaboration (36 %). Why are replication rates lower in the latter two projects? Possible explanations include the choice of *likely* versus *unlikely* replication candidates. Amongst the Many Labs studies, some had already previously been replicated and were selected knowing this fact. By contrast, the studies in the Pipeline project had not been previously replicated (indeed, not even previously published). Also important from a different perspective is whether each study was replicated only once by one group or multiple times by many groups.

In the Many Labs and Pipeline projects, 36 and 25 separate research groups were replicating each of 13 and 10 studies respectively. Multiple analyses lend themselves to meta-analytic techniques and analysis of the heterogeneity across research groups examining the same effect – the extent to which they accord in their effect sizes or not. The Many Labs project reported *I2* values, which estimate the proportion of variation due to heterogeneity rather than chance. In the majority of cases, heterogeneity was small to moderate or even non-existent (e.g. across the 36 replications for both of the social priming studies: flag and money). Indeed, heterogeneity of effect sizes was greater between studies than within studies. When heterogeneity was greater, it was - perhaps surprisingly - where mean effect sizes were largest. Nonetheless, Many Labs reassuringly shows that some effects are highly replicable across research groups, countries, presentational differences (online versus face to face).

Counter-intuitive and even fanciful psychological hypotheses are not necessarily more likely to be false, but believing them to be so may influence researchers– even implicitly – in terms of how replications are conducted. In their extensive literature search, Makel et al. [40] reported that most direct replications are conducted by authors who proposed the original findings. This raises the thorny question of who should replicate? Almost 50 years ago Bakan [2] sagely warned that “If an investigator attempts to replicate his own investigation at another time, he will inevitably be under the influence of what he has already done…He should challenge, for example, his personal identification with the results he has already obtained, and prepare himself for finding both novelty and contradiction with respect to his earlier investigation” and that “…If one investigator is interested in replicating the investigation of another investigator, he should carefully take into account the possibility of suggestion, or his willingness to accept the results of the earlier investigator. …He should take careful cognizance of possible motivation for showing the earlier investigator to be in error, etc. [p. 110].” The irony is that as psychologists, we should be acutely aware of such biases - we cannot ignore the psychology of replication in the replication of psychology.

## What are we replicating and why?

### The cheap and easy

Few areas of psychology have fallen under the replication lens and where they have, they are psychology’s equivalent to take-away meals – easy to prepare studies (e.g. often using online links to questionnaires). Hence, the focus has tended to be on studies from social and cognitive psychology, and not for example developmental or clinical studies, which are more prohibitive. Other notable examples exist such as cognitive neuropsychology, where the single case study has been predominant for decades – how can anyone recreate the brain injury and subsequent cognitive testing in a second patient?

### The contentious

We cannot assert that the totality– or even a representative sample - of psychology has been scrutinised for replication. We can also see why some may feel *targeted*– replication does not (and probably cannot) occur in a random fashion. The vast majority of psychological studies are overlooked. To date, psychologists have *targeted* the unexpected, the curious, and newsworthy findings; and largely within a narrow range of areas (cognitive and social primarily). As psychologists, the need to sample more widely ought to go without saying; and one corollary of this, is that it makes no sense to claim that *psychology* is in crisis.

Too often perhaps, psychologists have been attracted to replicating contentious topics such as social priming, ego-depletion, psychic ability and so on. Some high impact journals have become repositories for the attention-grabbing, strange, unexpected and unbelievable findings. This goes to the systemic heart of the matter. Hartshorne & Schachner [27] amongst many others have noted “…replicability is not systematically considered in measuring paper, researcher, and journal quality. As a result, the *current incentive structure rewards the publication of non-replicable findings*…” (p.3 my italics). This is nothing new in science, as the quest for scientific prestige has historically resulted in a conflict between the goals of science and the personal goals of the scientist (see [47]).

### The preposterous

*“If there is no ESP, then we want to be able to carry out null experiments and get no effect, otherwise we cannot put much belief in work on small effects in non-ESP situations. If there is ESP, that is exciting. However, thus far it does not look as if it will replace the telephone”* (Mosteller [41], p 396)

From the opposite perspective, Jim Coyne (this issue) maintains that psychology would benefit from some “…provision for screening out candidates for replication for which a consensus could be reached that the research hypotheses were improbable and not warranting the effort and resources required for a replication to establish this.” The frustration of some psychologists is palpable as they peruse apparently improbable hypotheses. Coyne’s concern echoes that of Edwards [18] who half a century ago similarly remarked, *“If a hypothesis is preposterous to start with, no amount of bias against it can be too great. On the other hand, if it is preposterous to start with, why test it*?” Edwards (p 402). How preposterous can we get? According to Simmons et al. [51], it is “…unacceptably easy to publish “statistically significant” evidence consistent with *any* hypothesis. (p. 1359). Indeed, they managed to show by manipulating what they describe as *researcher degrees of freedom* (e.g. ‘data-peeking’, deciding when to stop testing participants, whether to exclude outlying data points)*,* that people appear to forget their age and claim to be 1.5 years younger after listening to the Beatles song “When I’m 64”.

The fact that seemingly incredible findings can be published raises disquiet about the methods *normally* employed by psychologists and in some circles, this has inflated to concerns about psychology more generally. Within the methodological and statistical frameworks that psychologists *normally* operate, we have to face the unpalatable possibility that the *wriggle room* for researchers is – unacceptably large. Further, it is implicitly reinforced, as Coyne notes, by the actions of some journals as well as media outlets– and until that is adequately addressed, little will change.

### The negative

Interestingly, the four replication projects outlined above almost wholly neglected null findings. To date, replication efforts are invariably aimed at *positive* findings. Should we not also try to replicate null findings? Given the propensity for positive findings to become *nulls*, what is the likelihood of reverse effects in more adequately powered studies? The emphasis on replicating positive outcomes betrays the wider bias that psychologist have against null findings per se (Laws [36]). The overwhelming majority of *published* findings in psychology are positive (93.5 %: [54]) and the aversion to null findings may well be worse in psychology than other sciences [20]. Intriguingly, we can see a hint of this issue inthe OSC reproducibility project, which did include 3 %of sampled findings that were null initially - and whiletwo were confirmed as nulls, one did indeed become significant.As psychologists, we might ponder how the biasagainst publishing null findings finds a clear echo in the bias against replicating null findings.

## A conflict between belief and evidence

The *wriggle room* is fertile ground for psychologists to exploit the disjunction between belief and evidence that seems quite pervasive in psychology. As remarked upon by Francis “Contrary to its central role in other sciences,it appears that successful replication is sometimes not related to belief about an effect in experimental psychology. *A high rate of successful replication is not sufficient to induce belief in an effect* [8]*, nor is a high rate of successful replication necessary for belief* [22].” The Bem [8] study documented “experimental evidence for anomalous retroactive influences on cognition and affect” or in plain language…precognition. Using multiple tasks, and nine experiments involving over 1,000 participants, Bem had implausibly demonstrated that the performance of participants reflected what happened *after* they had made their decision. For example, on a memory test, participants were more likely to remember words that they were *later* asked to practise i.e. memory rehearsal seemingly worked back in time. In another task, participants had to select which of two curtains on a computer screen hid an erotic image, and they did so at a level significantly greater than chance, but not when the hidden images were less titillating. Furthermore, Bem and colleagues [7] later meta-analysed 90 previous studies to establish a significant effect size of 0.22.

Bem presents nine replications of a phenomenon and a large meta-analysis, yet we do not believe it, while other phenomena do not so readily replicate (e.g. bystander apathy [22]) but we do believe in them. Francis [23] bleakly concludes “*The scientific method is supposed to be able to reveal truths about the world, and the reliability of empirical findings is supposed to be the final arbiter of science; but this method does not seem to work in experimental psychology as it is currently practiced*.” Whether we believe in Bem’s precognition, social priming, or indeed, any published psychological finding – researchers are operating within the methodological and statistical *wriggle room*. The task for psychologists is to view these phenomena like any other scientific question i.e. in need of explanation. If they can close-down the wriggle room, then we might expect such curios and anomalies to evaporate in a cloud of nonsignificant results.

While some might view the disjunction between belief and evidence as ‘healthy skepticism’, others might also describe it as resistance to evidence or even anti-science. A pertinent example comes from Lykken [37] who described a study in which people who see frogs in a Rorschach test – ‘frog responders’ – were more likely to have an eating disorder [48] – a finding interpreted as evidence of harboring oral impregnation fantasies and an unconscious belief in anal birth. Lykken asked 20 clinician colleagues to estimate the likelihood of this ‘cloacal theory of birth’ before and after seeing Sapolsky’s evidence. Beforehand, they reported a *“…median value of 0.01, which can be interpreted to mean, roughly, ‘I don't believe it’*” and after being shown the confirmatory evidence “…*the median unchanged at 0.01. I interpret this consensus to mean, roughly, ‘I still don’t believe it.’”* (p. 151–152)*.* Lykken remarked that normally when a prediction is confirmed by experiment, we might expect “…a nontrivial increment in one’s confidence in that theory should result, especially when one’s prior confidence is low… [but that] this rule is wrong not only in a few exceptional instances *but as it is routinely applied to the majority of experimental reports in the psychological literature” p.152*. Often such claims give rise to a version of Feynman’s maxim that “Extraordinary claims require extraordinary evidence”. The *remarkableness* of a claim, however, is not necessarily relevant to either the type or the scale of evidence required. Instead of setting different criteria for the ordinary and extraordinary, we need to continue to close the *wriggle room*.

### Beliefs and the failure to self-correct

“Scientists should not be in the business of simply ignoring literature that they do not like because it contests their view.” [30]

Taking this to the opposite extreme, some researchers may choose to ignore the findings of meta-analyses at the expense of selected individual studies that accord more with their view. Giner-Sorolla [24] maintained that “…meta-analytic validation is not seen as necessary to proclaim an effect reliable. Textbooks, press reports, and narrative reviews often rest conclusions on *single influential articles rather than insisting on a replication across independent labs and multiple contexts*” (p 564, my italics).

Stoebe & Strack rightly point-out, “Even multiple failures to replicate an established finding would not result in a rejection of the original hypothesis, if there are also multiple studies that supported that hypothesis.” [and] ‘believers’ “…will keep on believing, pointing at the successful replications and derogating the unsuccessful ones, whereas the nonbelievers will maintain their belief system drawing on the failed replications for support of their rejection of the original hypothesis.” (p.64). Psychology rarely – if ever- proceeds with an unequivocal knock-out blow delivered by a negative finding or even a meta-analysis. Indeed, psychology often has more of the feel of trench warfare, where models and hypotheses are ultimately abandoned largely because researchers lose interest [26].

Jussim et al. [30] provide some interesting examples of precisely how social psychology doesn’t seem to correct itself when big findings fail to replicate. If doubts are raised about an original finding then as Jussim et al point out, we might expect citations to reflect this debate, the uncertainly and as such the original and the unsuccessful replications would be expected to be fairly equally cited.

In a classic study, Darley & Gross [15] found people applied a stereotype about social class when they saw a young girl taking a maths test either after seeing her playing in an affluent or poor background. After obtaining the original materials and following the procedure carefully, Baron et al. [6] published two failed replications using more than twice as many participants. Not only did they fail to replicate, the evidence was in the opposite direction. Such findings ought to encourage debate with relatively equal attention to the pro and con studies in the literature - alas no. Jussim et al. reported that “…since 1996, the original study has been cited 852 times, while the failed replications have been cited just 38 times (according to Google Scholar searches conducted on 9/11/15).”

This is not an unusual case, as Jussim et al. report several examples of failed replications not being cited, while original studies continue to be readily cited. The infamous and seminal study by Bargh and colleagues [5] showed that unconsciously priming people with an ‘elderly stereotype’ (unscrambling jumbled sentences that contained words like: *old, lonely, bingo, wrinkle*) makes them subsequently walk more slowly. However, Doyen et al. [16] failed to replicate the finding using more accurate measures of walking speed. Since 2013, Bargh et al. has been cited 900 times and Doyen et al. 192. Or a meta-analysis of 88 studies by Jost et al. [29] showing that conservativism is a syndrome characterized by rigidity, dogmatism, prejudice, and fear, not replicated by a larger better controlled meta-analysis conducted by Van Hiel and colleagues [57]. Since 2010, the former has been cited 1030 times while the latter a mere 60 by comparison. Jussim et al. suggest “This pattern of ignoring correctives likely leads social psychology to overstate the extent to which evidence supports the original study’s conclusions…[] it behooves researchers to grapple with the full literature, not just the studies conducive to their preferred arguments”.

### Meta-analysis: rescue remedy or statistical alchemy?

Some view meta-analysis as the closest thing we have to a definitive approach for establishing the veracity and reliability of an effect. In the context of discussing social priming experiments, John Bargh [4] declared that “…*In science the way to answer questions about replicability of effects is through statistical techniques such as meta-analysis*”. Others are more skeptical: “Meta-analysis is a reasonable way to search for patterns in previously published research. It has serious limitations, however, as a method for confirming hypotheses and for establishing the replicability of experiments” (p. 486 Hyman, 2010). Meta-analysis is not a magic dust that we can sprinkle over primary literatures to elucidate necessary truths. Likewise totemically accumulating replicated findings, in itself, does not necessarily prove anything (pace Popper). Does it matter if we replicate a finding once, twice, or 20 times, what ratio of positive to negative outcomes do we find acceptable? Answers or rules of thumb do not exist – it often comes down to our *beliefs* in psychology.

This special issue of *BMC Psychology* contains 4 articles (Taylor & Munafo, [56]; Lakens, Hilgaard & Staaks [34]; Coppens, Verkoeijen, Bouwmeester & Rikers, [13]; Coyne [14]) and in each, meta-analysis occupies a pivotal place. As shown by Taylor & Munafo (current issue), meta analyses have proliferated, are highly cited and “…most worryingly, the perceived authority of the conclusions of a meta-analysis means that it has become possible to use a meta-analysis in the hope of having the final word in an academic debate.” As with all methods, meta-analysis has its own limitations and *retrospective* validation via meta-analysis is not a substitute for *prospective* replication using adequately powered trials, but they do have substantive role to play in the reproducibility question.

Judging the weight of evidence is never straightforward and whether a finding sustains in psychology often reflect our beliefs almost as much as the evidence. Indeed, meta-analysis rightly *or* wrongly enables some ideas to persist despite a lack of support at the level of individual study or trial. This has certainly been argued in the use of meta-analyses to establish a case for psychic abilities, where Storm, Tressoldi & Di Risio [55] identify how “It distorts what scientists mean by confirmatory evidence. It confuses retrospective sanctification with prospective replicability.” (p.489)

This is a kind of free-lunch’ notion of meta-analysis. Feinstein [21] even stated that “*meta-analysis is analogous to statistical alchemy for the 21st century*…*the main appeal is that it can convert existing things into something better. “Significance” can be attained statistically when small group sizes are pooled into big ones”* (p. 71). Undoubtedly, the conclusions of meta-analyses may prove unreliable where small numbers of nonsignificant trials are pooled to produce significant effects [19]. Nonetheless, it is also quite feasible for a majority of negative outcomes in a literature and still produce a reliable overall significant effect size (e.g. streptokinase: [35]).

Two of the papers presented here (Lakens et al. this issue; Taylor & Munafo this issue) offer extremely good suggestions relating to some of these conflicts in meta-analytic findings. Lakens and colleagues offer 6 recommendations, including permitting others to “re-analyze the data to examine how sensitive the results are to subjective choices such as inclusion criteria” and enabling this by providing links to data files that permit such analysis. Currently, we also need to address data sharing in regular papers. Sampling papers published in one year in the top 50 high-impact journals, Alsheikh-Ali et al. [1] reported that a substantial proportion of papers published in high-impact journals “…are either not subject to any data availability policies, or do not adhere to the data availability instructions in their respective journals”. Such efforts for transparency are extremely welcome and indeed, echo the posting online of our interactive CBT for schizophrenia meta-analysis database (http://www.cbtinschizophrenia.com/), which has been used by others to test new hypotheses (e.g. [25]).

Taylor & Munafo (this issue) advise greater *triangulation* of evidence and in this particular instance, supplementing traditional meta-analysis and P-curve analysis [52]. In passing, Taylor & Munafo also mention “…adversarial collaboration, where primary study authors on both sides of a particular debate contribute to an agreed protocol and work together to interpret the results”. The proposed version of adversarial collaboration proposed by Kahneman [31] urged scientists to engage in a “good-faith effort to conduct debates by carrying out joint research” (p. 729). More recently, he elaborated on this in the context of the furore over failed replications (Kahneman [32]). Coyne covers some aspects of this latest paper on replication etiquette and finds some of it wanting. It may however be possible to find some new adversarial middle ground, but it crucially depends upon psychologists being more open. Indeed, some aspects of *adversarial collaboration* could dovetail with Lakens et als’ proposal regarding hosting relevant data on web platforms. In such a scenario, opposing views could test their hypotheses in a public arena using a shared database.

In the context of adversarial collaboration, some uncertainty and difference of opinion exists about how we might accommodate the views of those being replicated. One possibility again requires openness and that is for those who are replicated to be asked to submit a review; and crucially, the review and replicator’s responses are then published alongside the paper. Indeed, this happened with the paper of Coppens et al. (this issue). They replicated the ‘testing effect’ reported by Carpenter (2009) – that information which has been retrieved from memory is better recalled than that which has simply been studied. Their replications and meta-analysis partially replicate the original findings, and Carpenter was one of the reviewers whose review is available alongside the paper (along with the author responses). Indeed, from its initiation, BMC Psychology has published all reviews and responses to reviewers alongside published papers. This degree of *openness* is unusual in psychology journals, but does offer readers a glimpse into the process behind a replication (or any paper), allows the person being replicated to contribute and comment on the replication, to reply and be published in the same journal at the same time.

Ultimately, the issues that psychologists face over replication are as much about our beliefs, biases and openness as anything else. We are not dispassionate about the outcomes that we measure. Maybe because the *substance* of our spotlight is people, cognition and brains, we sometimes *care* too much about the ‘truths’ we choose to declare. They have implications. Similarly, we should not ignore the incentive structures and conflicts between the personal goals of psychologists and the goals of science. They have implications. Finally, the attitudes of psychologists to the transparency of our science needs to change. They have implications.
